# Quantitative Evaluation of a Cross-Sectional Area of the Fetal Straight Sinus by Magnetic Resonance Imaging and Its Clinical Value

**DOI:** 10.3389/fneur.2022.875402

**Published:** 2022-07-22

**Authors:** Shi-Jia Fu, Jing-Bo Xu, Xin Liu, Yi-Min Cao, Ze-Xi Yi, Li-Xia Zhou

**Affiliations:** Department of Medical Imaging, The Second Hospital of Hebei Medical University, Shijiazhuang, China

**Keywords:** fetus, straight sinus, magnetic resonance imaging, gestational age, medical imaging

## Abstract

**Objective:**

The intracranial venous system plays an important role in ensuring blood circulation and a stable blood supply to the fetal brain. In the present study, a cross-sectional area of the fetal straight sinus was quantitatively evaluated by fetal magnetic resonance imaging (MRI) to explore the method's clinical value.

**Methods:**

The clinical and MRI data of 126 normal fetuses in mid-to-late stage pregnancies were retrospectively analyzed. The “dominant” sequence of the fetal straight sinus was selected, and the cross-sectional area of the lumen was measured at each gestational age to obtain the normal range at different ages and to analyze the developmental pattern and characteristics of the fetal straight sinus.

**Results:**

There were statistically significant differences in the cross-sectional area of the fetal straight sinus among different gestational ages (*P* < 0.05). The cross-sectional area of the fetal straight sinus was positively correlated with gestational age (coefficient of determination = 0.6892, *P* < 0.05). That is, the cross-sectional area of the fetal straight sinus grew with increasing gestational age, and the regression equation was *y* = 0.27 *x* – 2.14 (*P* < 0.05). Additionally, there were five fetuses with cerebral venous abnormalities, including four with heart failure and one with venous sinus thrombosis.

**Conclusion:**

Quantitative measurement of a cross-sectional area of the fetal straight sinus by MRI enhanced understanding of the anatomical features and developmental pattern of fetal cerebral veins and provided a reference for the clinical diagnosis of related diseases and investigation concerning pathogenesis.

## Introduction

Fetal cerebral blood circulation is vital for cranial cerebral development and has become a popular area of research. Abnormalities in cerebral circulation during the fetal stage may cause hypoxia, intrauterine growth restriction, neurological impairment, and other disorders (1). Improving an understanding of normal fetal cerebral blood circulation is important for promoting the investigation of fetal brain pathophysiology and cerebrovascular malformations (2). The intracranial venous system plays a vital role in the cerebral circulation system, and the straight sinus is the main pathway for deep cerebral venous blood return, thereby fulfilling an important role in this system (3).

The biological indicators of the straight sinus are important parameters for assessing fetal cranial development and screening for cranial anomalies. Objective quantitative analysis of the cross-sectional area of the straight sinus, based on a large sample of normal fetuses at different stages of development, has to date not been reported. Fetal intracranial veins can be visualized with high-resolution Doppler ultrasound equipment; however, the technique's image quality is not ideal, based on limitations caused by a reduced volume of amniotic fluid, maternal obesity, special fetal position, fetal skull ossification, and the maternal pelvis (4, 5). The fetal magnetic resonance imaging (MRI) technique, an essential adjunct to prenatal fetal examination, has a superior ability for visualizing fetal vascular structures. In the present study, fetal MRI was adopted to quantitatively evaluate the luminal cross-sectional area of the straight sinus in normal fetuses at different developmental stages. The approach was used to investigate developmental characteristics during the fetal stage and provide a reference for basic research and the clinical diagnoses of related diseases.

## Materials and Methods

### General Data

Pregnant women who underwent MRI due to suspected fetal intracranial lesions by prenatal ultrasound at the Second Hospital of the Hebei Medical University (China) from October 2018 to August 2020 were retrospectively analyzed. In total, 126 patients with normal cranial structures, who had been diagnosed by MRI and confirmed by postnatal follow-up, were randomly enrolled in the present study. The maternal age ranged from 20 to 39 years, with an average age of 29.5 ± 5.6 years, and the gestational ages ranged from 19 to 38 weeks, with an average age of 28.5 ± 5.6 weeks. Five fetuses with cerebral venous abnormalities, including 4 with heart failure and 1 with venous sinus thrombosis received MRI analysis.

- The study's inclusion criteria were as follows: a singleton pregnancy with a definite gestational age; no neurological or intracranial venous lesions in the fetus; maternal age >18 years; gestational age >13 weeks and <40 weeks.
- The study's exclusion criteria were as follows: the quality of the MRI failed to meet the requirements for quantitative assessment; pregnant women with chronic diseases, such as hypertension, diabetes mellitus, kidney disease, or genetic syndromes.

### Methods

An Optima MR360 1.5T MR (General Electric, USA) scanner was used to perform MR plain scanning of the fetal head and neck, and axial, coronal, and sagittal images were acquired. The pregnant woman was placed in a supine position, feet first, while breathing calmly. The scanning sequence and parameters were as follows: fast imaging employing a steady-state acquisition (FIESTA) sequence with a repetition time/time to echo (TR/TE) of 5.3/2.3 ms; a single shot-fast spin-echo (SS-FSE) sequence with a TR/TE of 2,000/140 ms; a fast inversion recovery motion insensitive (FIRM) sequence with a TR/TE of 150/4.2 ms; a diffusion-weighted imaging (DWI) sequence with a TR/TE of 3,274/75 ms, *b* = 0, 700 s/mm^2^; the layer thickness was 5 mm, and field of vision parameters were 380 × 380 mm.

### Image Analysis and Post-processing

**Figure 1:**
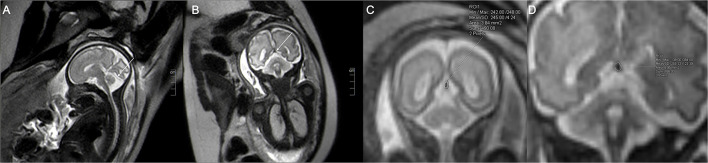
The single shot-fast spin-echo sequence of a normal fetus at a gestational age of 32 weeks. The sagittal plane **(A)** shows the intact straight sinus (arrow), and the coronal plane **(B)** shows the cross-section of the fetal straight sinus (arrow) with measurement of the cross-sectional area. **(C)** (image magnification, × 8) and **(D)** (image magnification, × 10) show 23 and 31-week fetuses, respectively. The measurement method of straight sinus and the measured sectional area are shown in the figure.

**Figure 2:**
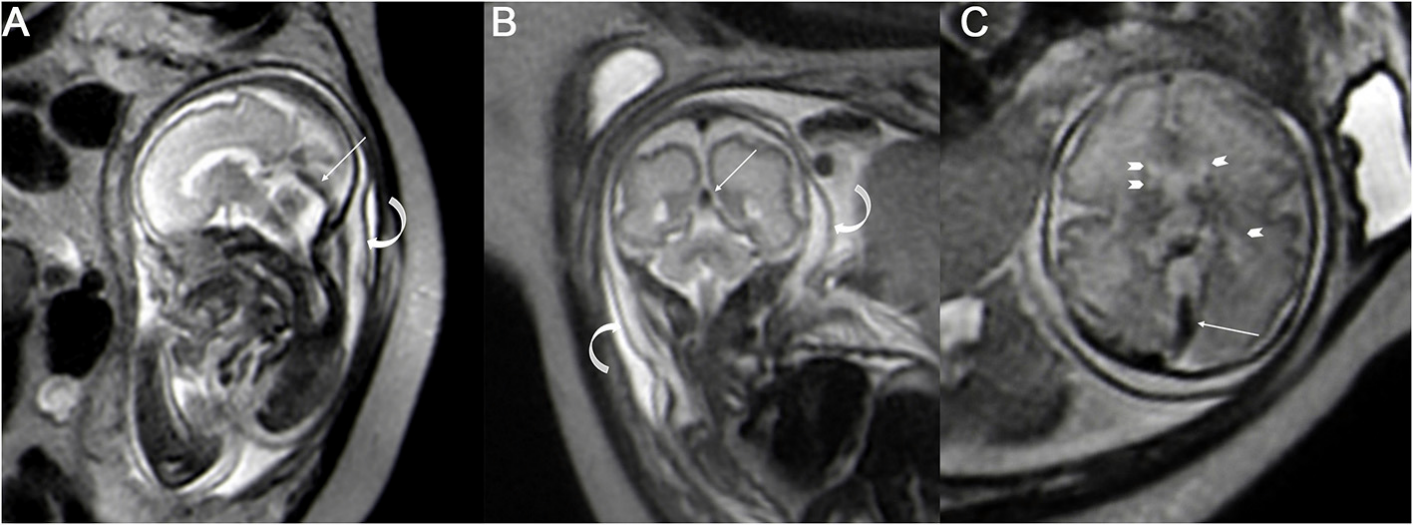
The single shot-fast spin-echo sequence for a fetus with heart failure at a gestational age of 30 weeks; the sagittal plane **(A)** and coronal plane **(B)** and axial plane **(C)** show the dilated straight sinus (straight arrow), and the extensive subcutaneous edema (**A**,**B** curved arrow) and sub-ependymal cysts can be seen around the bilateral lateral ventricles (**C** arrowhead).

The fetal MRIs were imported into Siemens workstations for analysis and recording measurements. The fetal cephalogram was viewed in the median sagittal plane. The short vein of Galen, which runs posteriorly and superiorly, was first found immediately below the splenium of the corpus callosum. The vein of Galen turned downward and continued into a thicker and more pronounced vein, which ran downward and backward along the tentorium cerebelli to the sinus confluence in the occipital region of the skull, i.e., the straight sinus (Figure 1A). Then, the contours of the straight sinus lumen (excluding the wall) were manually outlined in the oblique coronal plane (Figure 1B) and automatically computer-processed to generate the cross-sectional area image (Figures 1C,D). Measurement was conducted three times, and the average was taken as the final result. Two senior imaging physicians performed the work independently, and the patients' clinical information was hidden during the image measurement. The straight sinuses of five fetuses with abnormal cerebral veins were dilated to varying degrees, and the cross-sectional area exceeded the normal range. The fetus with heart failure showed that the deep veins in the brain, particularly the lumen of the straight sinus, were significantly thickened. With the presence of fetal scalp edema (Figures 2A–C), and the formation of sub-ependymal cysts in bilateral lateral ventricles (Figures 1C,D). Additionally, the venous sinus thrombus straight sinus showed thickening with a thrombus signal.

### Statistical Analysis

The SPSS Statistics 26.0 software program was adopted to perform statistical analysis. The measurement data were expressed as mean ± standard deviation ($\bar{x}$ ± *s*), and *P* < 0.05 was considered statistically significant. One-way ANOVA was performed to calculate the 95% confidence intervals for the cross-sectional area of the fetal straight sinus at different gestational ages, and two-by-two comparisons were made. Regression analysis was conducted between the cross-sectional area of the fetal straight sinus and gestational ages.

## Results

The measurement results of the cross-sectional area of the fetal straight sinus at each gestational age are summarized in Table 1. The cross-sectional area of the fetal straight sinus was normally distributed with equal variance. The ANOVA results showed statistically significant differences (*P* < 0.05).

**Table 1:** The measurement of the cross-sectional area of the fetal straight sinus.

**The gestational age (*W*)**	**The number of cases (*n*)**	**The cross-sectional area of the fetal straight sinus (mm** ^**2**^ **)**		
		**±*s***	**Median**	**95% CI**
19	3	2.98 ± 0.43	3.25	(2.14, 3.82)
20	3	3.26 ± 0.48	3.38	(2.80, 3.72)
21	4	3.48 ± 0.49	3.62	(2.52, 4.44)
22	4	3.83 ± 1.28	3.80	(1.32, 6.34)
23	6	4.15 ± 0.75	4.06	(2.68, 5.62)
24	6	4.42 ± 0.44	4.45	(3.56, 5.28)
25	4	4.58 ± 0.53	4.64	(3.54, 5.62)
26	8	4.87 ± 1.17	5.00	(2.58, 7.16)
27	8	5.33 ± 0.80	5.12	(3.76, 6.90)
28	12	5.49 ± 0.97	5.69	(3.59, 7.39)
29	10	5.78 ± 0.32	5.83	(5.47, 6.08)
30	12	5.87 ± 0.11	6.14	(5.65, 6.09)
31	11	6.38 ± 0.95	6.48	(4.52, 8.24)
32	10	6.57 ± 0.63	6.36	(5.34, 7.80)
33	6	6.84 ± 0.42	6.84	(6.02, 7.66)
34	4	6.96 ± 0.90	7.11	(5.20, 8.72)
35	4	7.11 ± 1.26	6.82	(4.64, 9.58)
36	4	7.42 ± 0.37	7.29	(6.69, 8.15)
37	4	7.95 ± 0.93	8.42	(6.13, 9.77)
38	3	8.44 ± 1.04	7.95	(6.40, 10.48)

*95% CI, the 95% confidence interval*.

**Figure 3:**
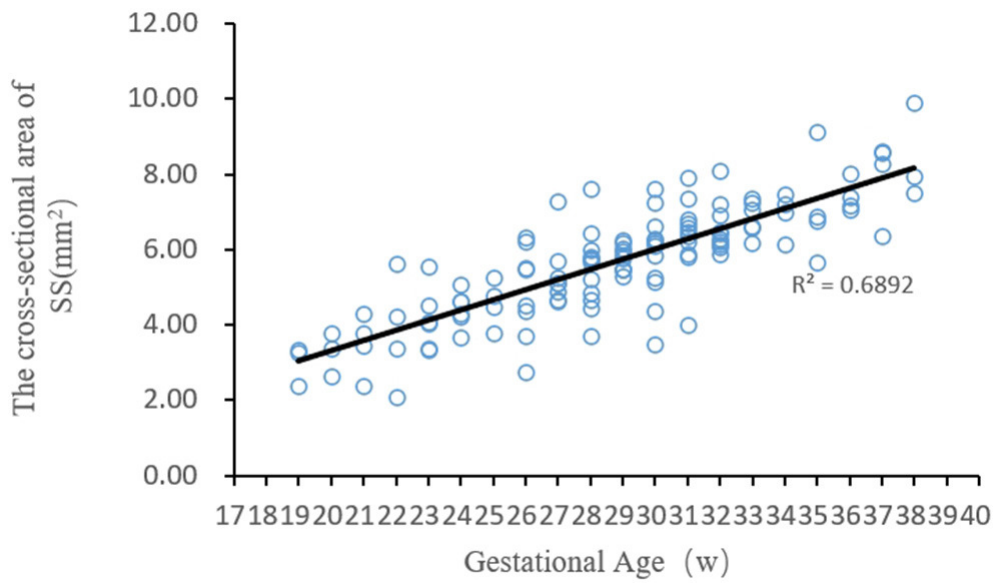
The fitting curve between the cross-sectional area of the fetal straight sinus at different gestational ages and the gestational age.

The regression analysis results showed that the cross-sectional area of the fetal straight sinus positively correlated with the gestational age, i.e., it increased with gestational age. Curve-fitting was performed between the cross-sectional areas of the fetal straight sinus at different gestational ages (Figure 3), and its regression equation vs. gestational age was obtained as *y* = 0.27 *x* − 2.14.

## Discussion

Fetal MRI is becoming a widely accepted method of prenatal screening. In the past three decades, with the popularization of rapid MRI technology, it has become possible to obtain a high-quality image of the fetus without sedation, thereby improving the visualization of fetal anatomical details and compensating for the drawbacks of prenatal ultrasound imaging (5). Because the deep venous drainage system is relatively immature in the developing brain, some reports suggest that fetal deep cerebral vein dilatation may be related to fetal brain injury, including perivenous hemorrhage and various types of brain edema, the common cause of which is heart failure (6). In the current study, the findings for fetal straight sinus dilatation and the presence of a sub-ependymal cyst in central force failure closely matched the findings of the above-noted study.

The accurate measurement of fetal cerebral veins is essential for assessing fetal cerebral circulation and cranial development. The advantages of high-resolution MRI for visualizing small fetal vessels make measuring fetal cerebral veins possible. Researchers have recognized the serious outcomes of abnormalities in the fetal venous system, but research in this area has to date been limited to a description of the imaging features in individual cases, and no general pattern of the fetal cranial venous system has been established. Accordingly, there is an increasingly urgent need for standardizing MRI data (6, 7).

In the present study, a cross-sectional area of the fetal straight sinus was quantified and evaluated by drawing on the common indicators of fetal coronary veins in prenatal ultrasonography during mid-to-late-term pregnancy; (8) this process assisted in establishing a normal reference range and providing a reference basis for the diagnosis of fetal developmental abnormalities/lesions as a means to prevent or mitigate fetal brain parenchymal injury caused by venous-related diseases.

In the present study, an SS-FSE sequence was applied to quantitatively assess the fetal straight sinus. The commonly used classifications for fetal MRI include SS-FSE, FIESTA, FIRM, and DWI sequences (9). To ensure the accuracy and reproducibility of the results, the sequences adopted in the present study had to provide the desired contrast at all gestational ages. The high signal in the cerebrospinal fluid gap around the brain, and the low signal in the lumen of the intracranial vessels (due to the “flow-through” effect in the SS-FSE sequence) formed an appropriate contrast, and the high resolution of this sequence facilitated the visualization of the fetal straight sinus. These findings were consistent with research conducted by Masselli et al. (10).

In the FIESTA sequence, the clarity of the fetal brain structures was fair, but the display of intracranial vessels was limited. The FIRM sequence involved T1-weighted imaging with an insufficient contrast of each structure and a low signal-to-noise ratio, and the structure of the fetal straight sinus could not be clearly visualized. This may have been due to the extended scanning time required for this sequence, which could easily have been affected by fetal movement. In existing studies, the FIRM sequence was more commonly used to diagnose fetal cerebral hemorrhage (11). Although DWI sequences had significant advantages in investigations evaluating fetal brain development (10), this sequence was observed to have had some amplification effect on the fetal straight sinus in the present study. Therefore, the SS-FSE sequences may be more suitable than others for observing and evaluating the fetal straight sinus.

The results of the present study showed a positive correlation between the cross-sectional area of the fetal straight sinus and gestational age, which was considered to be related to the development of fetal brain tissue and changes in the blood flow of drainage channels. In mid-to-late gestational age, the primitive forebrain's central vein degenerated and disappeared, leaving the vein of Galen and the straight sinus as the most critical deep vein drainage channels (11). Additionally, as the brain volume increased with growth and development during the fetal stage, the volume of cerebral blood circulation also increased (12). As such, the amount of venous blood draining from the straight sinus increased in relative and absolute terms during fetal growth and development in mid-to-late pregnancy. The luminal cross-sectional area of the straight sinus increased as the fetus grew and developed, commensurate with the role it plays. Ma et al. (8) measured the coronary veins of the fetal heart using ultrasound and found that the internal diameter of the fetal coronary veins increased progressively with the duration of gestation, which, to some extent, provided parallel corroboration of the present study's findings.

The quantitative assessment of the normal fetal straight sinus in the present study yielded its relevant parameters and general developmental characteristics, which may be useful as a reference for the diagnosis of prenatal abnormalities of the cerebral venous system. The most common disease of the cerebral venous system in fetuses is an aneurysmal malformation of the vein of Galen, which is often detected in the neonatal period and frequently causes dilatation of the vein of Galen and the straight sinus (4). Fetuses with this malformation often develop epilepsy or high-output heart failure (12), and even fetal edema (13). In fetuses with growth restriction, increased blood flow due to an increased oxygen demand in the brain, as well as the redistribution of blood flow, results in an increased cerebral venous return and dilatation of the cerebral veins (14). Dilatation of the fetal straight sinus often causes stagnant blood flow and may be combined with cerebral venous thrombosis (14, 15).

Currently, there is a limited understanding of the causes and long-term clinical significance of fetal dural sinus dilatation combined with thrombosis; furthermore, smaller thrombi in the fetal straight sinus are not always easily distinguishable from intra-arachnoid granules in the straight sinus (15, 16). The quantitative data on the cross-sectional area of the fetal straight sinus in the present study may be useful for making a differential diagnosis between these two aspects. Fetal hypoplasia or atresia of the straight sinus may result in degenerative failure and the formation of a permanent falciform vein (17).

Straight sinus stenosis in adulthood is believed to occur due to non-aneurysmal hemorrhage in the midbrain area (18, 19), but there are no relevant existing studies on this aspect in the fetal stage. Therefore, quantitative MRI assessment of the cross-sectional area of the fetal straight sinus may be able to better evaluate the degree of its dilatation or stenosis, provide more detailed prenatal diagnostic information, facilitate the diagnosis and assessment of the prognosis of the affected fetus, and provide timely targeted treatment for the newborn.

There were some limitations in the present study. The sample size was limited and the correlation between abnormalities in the straight sinus measurements and developmental lesions of the venous system or brain during the fetal stage remains to be clarified, with the exception of a few diseases. Because of the uneven distribution of classes according to GA (the majority of cases restricted between 26 and 32 weeks), the results of a study with more evenly distributed cases might present some differences from our study.

## Conclusion

In conclusion, the objective and quantitative assessment of the cross-sectional area of the fetal straight sinus by MRI, and the establishment of a standard reference will help to provide a better understanding of the development of the fetal venous system; additionally, doing so may also help to identify abnormalities in this area, provide new insights into the pathological mechanisms of fetal-related diseases, and address the gap in knowledge related to the research on the fetal straight sinus.

## Data Availability Statement

The raw data supporting the conclusions of this article will be made available by the authors, without undue reservation.

## Ethics Statement

This study was conducted with approval from the Ethics Committee of Second Hospital of Hebei Medical University (No: 2016164). Written informed consent to participate in this study was provided by the participants' legal guardian/next of kin.

## Author Contributions

Conception and design of the research: L-XZ. Acquisition of data: S-JF and XL. Analysis and interpretation of the data: J-BX. Statistical analysis: S-JF. Writing of the manuscript: Y-MC. Critical revision of the manuscript for intellectual content: Z-XY. All authors read and approved the final draft.

## Conflict of Interest

The authors declare that the research was conducted in the absence of any commercial or financial relationships that could be construed as a potential conflict of interest.

## Publisher's Note

All claims expressed in this article are solely those of the authors and do not necessarily represent those of their affiliated organizations, or those of the publisher, the editors and the reviewers. Any product that may be evaluated in this article, or claim that may be made by its manufacturer, is not guaranteed or endorsed by the publisher.
